# Estimating the Number of COVID-19 Cases and Impact of New COVID-19 Variants and Vaccination on the Population in Kerman, Iran: A Mathematical Modeling Study

**DOI:** 10.1155/2022/6624471

**Published:** 2022-04-26

**Authors:** Mehran Nakhaeizadeh, Maryam Chegeni, Masoumeh Adhami, Hamid Sharifi, Milad Ahmadi Gohari, Abedin Iranpour, Mahdieh Azizian, Mashaallah Mashinchi, Mohammad Reza Baneshi, Mohammad Karamouzian, Ali Akbar Haghdoost, Yunes Jahani

**Affiliations:** ^1^Modeling in Health Research Center, Institute for Futures Studies in Health, Kerman University of Medical Sciences, Kerman, Iran; ^2^Department of Biostatistics and Epidemiology, School of Public Health, Kerman University of Medical Sciences, Kerman, Iran; ^3^Department of Basic and Medical Laboratory Sciences, Khomein University of Medical Sciences, Khomein, Iran; ^4^Molecular and Medicine Research Center, Khomein University of Medical Sciences, Khomein, Iran; ^5^HIV/STI Surveillance Research Center, and WHO Collaborating Center for HIV Surveillance, Institute for Futures Studies in Health, Kerman University of Medical Sciences, Kerman, Iran; ^6^Department of General Educations, Afzalipour School of Medicine, Kerman University of Medical Sciences, Kerman, Iran; ^7^Department of Statistics, Faculty of Mathematics and Computer, Shahid Bahonar University of Kerman, Kerman, Iran; ^8^School of Public Health, The University of Queensland, Brisbane, Queensland, Australia; ^9^Brown School of Public Health, Brown University, Providence, RI, USA; ^10^Social Determinants of Health Research Center, Institute for Futures Studies in Health, Kerman University of Medical Sciences, Kerman, Iran

## Abstract

COVID-19 is spreading all over Iran, and Kerman is one of the most affected cities. We conducted this study to predict COVID-19-related deaths, hospitalization, and infected cases under different scenarios (scenarios A, B, and C) by 31 December 2021 in Kerman. We also aimed to assess the impact of new COVID-19 variants and vaccination on the total number of COVID-19 cases, deaths, and hospitalizations (scenarios D, E, and F) using the modified susceptible-exposed-infected-removed (SEIR) model. We calibrated the model using deaths reported from the start of the epidemic to August 30, 2021. A Monte Carlo Markov Chain (MCMC) uncertainty analysis was used to estimate 95% uncertainty intervals (UI). We also calculated the time-varying reproductive number (*R*_t_) following time-dependent methods. Under the worst-case scenario (scenario A; contact rate = 10, self‐isolation rate = 30%, and average vaccination shots per day = 5,000), the total number of infections by December 31, 2021, would be 1,625,000 (95% UI: 1,112,000–1,898,000) with 6,700 deaths (95% UI: 5,200–8,700). With the presence of alpha and delta variants without vaccine (scenario D), the total number of infected cases and the death toll were estimated to be 957,000 (95% UI: 208,000–1,463,000) and 4,500 (95% UI: 1,500–7,000), respectively. If 70% of the population were vaccinated when the alpha variant was dominant (scenario E), the total number of infected cases and deaths would be 608,000 (95% UI: 122,000–743,000) and 2,700 (95% UI: 700–4,000), respectively. The *R*_t_ was ≥1 almost every day during the epidemic. Our results suggest that policymakers should concentrate on improving vaccination and interventions, such as reducing social contacts, stricter limitations for gathering, public education to promote social distancing, incensing case finding and contact tracing, effective isolation, and quarantine to prevent more COVID-19 cases, hospitalizations, and deaths in Kerman.

## 1. Introduction

SARS-CoV-2 and its newly emerging variants continue to spread worldwide [1]. As of November 3, 2021, a total number of 248,590,953 COVID-19 cases and 5,034,002 COVID-19-related deaths have been reported globally [2]. Iran is one of the most affected countries by COVID-19. The first confirmed cases of COVID-19 in Iran were reported on February 19, 2020 [3], and since then, COVID-19 has spread rapidly across the country. Until November 3, 2020, the Iranian Ministry of Health and Medical Education has reported more than 5,954,962 confirmed COVID-19 cases and 126,763 COVID-19-related deaths in Iran [4]. Kerman Province, located in the southeast of the country, is the largest province of Iran, with a population of about 3 million people [5]. The first two cases of COVID-19 in Kerman were identified in early March 2020 [6]. During the following months, COVID-19-related hospitalized cases increased to 40,765, and COVID-19-related deaths reached 4,825 in the province [7].

Upon the onset of the COVID-19 epidemic in Iran, the government started implementing various nonpharmaceutical interventions (NPIs), such as social distancing, closure of universities and schools all over the country, reduction of official work hours, limiting public gatherings, restriction of travel to or from cities with the high case numbers, and extensive lockdown of high-risk occupations [8–10]. Active contact tracing and increasing testing on outpatients have also been performed to prevent the spread of COVID-19. Moreover, all patients were trained on self-isolation principles and home-based care.

The emergence of different SARS-CoV-2 variants has posed challenges to epidemic control efforts in Iran. In early June 2021, the delta variant spread throughout Iran, especially in Kerman's southern neighbouring provinces (e.g., Sistan and Baluchistan) [11, 12]. Although the effectiveness of vaccines to symptomatic infection against emergent variants of the virus (e.g., delta) is decreasing (>70% compared to the alpha variant) [13], vaccines have shown good efficacy against severe disease, hospitalization, and death. In February 2021, the vaccination program was implemented, prioritizing older adults and health care workers in Iran. As of November 20, 2021, 44.7 million people (53.2% of the total population) have been fully vaccinated [14]. A range of different COVID-19 vaccines, including Sinopharm vaccine, Oxford–AstraZeneca vaccine, COVIran Barekat, Sputnik V, PastoCovac, and Covaxin, have been administered in Iran [11]. The vaccination program in Kerman was initiated in mid-February 2021, and as of November 3, 2021, approximately 1,945,600 (61.4% of the total population) had received at least one dose of the vaccine, and 1,260,475 (39.8% of the total population) had received the second dose [7].

Dynamic mathematical models are effective tools for health policymakers to understand the transmission mechanisms of COVID-19 and predict COVID-19-related new cases and deaths [15]. The susceptible-exposed-infected-removed (SEIR) model is a forecasting model widely adopted to estimate epidemiological parameters of infectious diseases [16–18]. SEIR has been frequently used for modeling COVID-19 [10, 19–21]. For instance, deterministic SEIR and travel network-based SEIR models were used to assess the impact of various NPIs against COVID-19 [22–24]. Moreover, the SEIR model was used to estimate the transmission risk of COVID-19 and the effectiveness of contact tracing followed by quarantine and isolation in China [25]. Stochastic SEIR models have also been used to assess the impact of contact tracing and isolation on COVID-19 [26]. After the arrival of vaccination and new variants, mathematical models were developed to evaluate the impact of the vaccination and new variants on the population. For example, mathematical models have helped assess the impact of SARS-CoV-2 variants and vaccination campaigns on the spread of COVID-19 [27, 28]. Furthermore, mathematical models have illustrated COVID-19 transmission mechanism with vaccination [29] and helped assess the impact of NPIs, vaccination, and the SARS-CoV-2 delta variant in England [30]. Several studies have also calculated the time-varying effective reproduction number to evaluate the effectiveness of NPIs in several studies [31–34].

We modified a SEIR model for COVID-19 epidemic parameters to estimate the epidemic trend in the near future and the peak date under various epidemic scenarios in Iran's Kerman Province. We aimed to quantify timely vaccination programs, NPIs implemented by the government, and rate of self-isolation observed by patients and investigate the impact of three SARS-CoV-2 variants. Consequently, the aim of the current study was twofold: first, to predict Kerman's COVID-19-related deaths, hospitalization, and infected cases under different scenarios by December 31, 2021, and second, to assess the impact of new COVID-19 variants and vaccination on infected cases, death toll, and hospitalizations. The results of the model may provide a basis for public health practitioners and policymakers in adopting and promoting policies and strategies to prevent the rapid spread of COVID-19 in Kerman and potentially other parts of the country.

## 2. Methods

### 2.1. Mathematical Model

**Figure 1:**
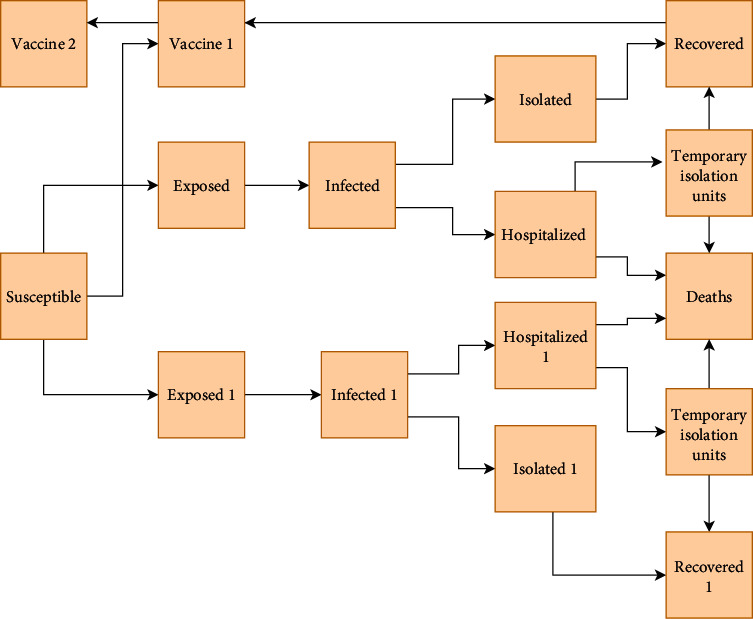
The SEIR conceptual model. Susceptible individuals who have not received vaccine and are not infected go to the exposed compartment. However, reinfected individuals or people who received the vaccine go to exposed 1 compartment.

A modified SEIR compartmental model was used to predict the total number of infected cases, deaths, and hospitalizations. We used the modeling framework based on previous work in Iran [35]. The model was calibrated based on epidemiological and clinical data from Kerman. We used the SEIRDI_s_HTV_1_V_2_ model with the following compartments: susceptible (S), latent (E), infected (I), recovered (R), deaths (D), isolated (I_s_), hospitalized (H), temporary isolation units (T), at least one dose vaccination (V_1_), and fully vaccinated (V_2_) (Figure 1). Susceptible individuals were those at risk of becoming infected by SARS-CoV-2. In our model, the total population of Kerman (3,165,000) was assumed as susceptible [5]. Infected people could transmit the infection after exposure to SARS-CoV-2 and during the latency period. However, exposed individuals could transmit the infection to susceptible individuals after the latency period (assuming the latent period and incubation period to be equal). We considered that infected individuals could be classified into four stages: (a) were asymptomatic or had mild symptoms and did not isolate themselves to recover after the infectious period, (b) were asymptomatic or had mild symptoms and self-isolated after showing clinical symptoms then recovered, (c) had severe symptoms and needed to go to the hospital after the onset of COVID-19 symptoms, and (d) died without being hospitalized. Moreover, some hospitalized individuals may die, and some admitted to temporary isolation units may recover.

It was assumed that a proportion of recovered and susceptible individuals belong to partially vaccinated (i.e., people who received at least one dose of vaccine) and then to the fully vaccinated compartment (i.e., received two doses). Furthermore, we assumed that people who received the vaccine could be infected again with other variants; however, the rate of death and hospitalization was lower than those who did not receive a vaccine. The vaccines used in Iran are mostly Sinopharm and AstraZeneca. The effectiveness of one dose of vaccine was assumed 50%, and the effectiveness of two doses was 70% [13]. In June 2021, the delta variant entered Kerman and spread across the province. On August 18 alone, about 32 people died in the province, which was the highest number of COVID-19-related deaths since the beginning of the pandemic [7]. In the model, we considered the effect of the delta variant from June 10, 2021 [12]. A proportion of recovered individuals would proceed into the susceptible group due to reinfection risk with the delta variant [36]. However, we assumed that the mortality rate and hospitalization rate for reinfection were lower for people who were not infected. The differential equations for each compartment are described in the Supplementary Material (Appnedix 1).

### 2.2. Calibration and Parameters

**Figure 2:**
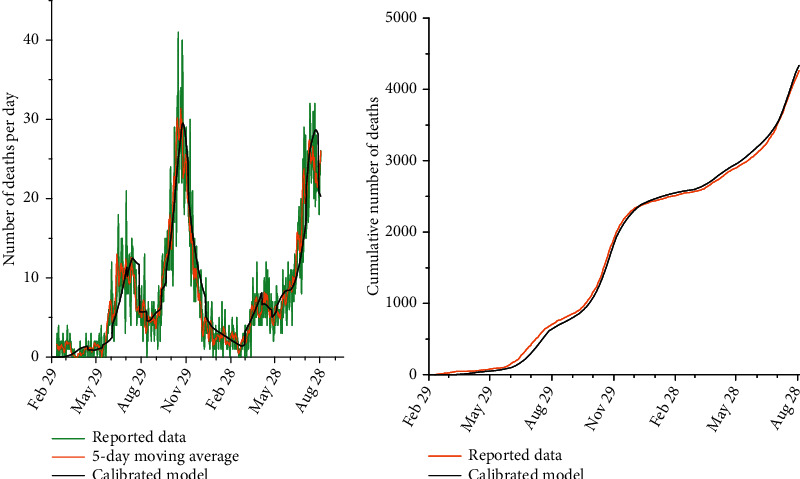
(a) Plots of the number of deaths per day versus different dates. The green line in this plot reports daily deaths in Kerman, the orange line shows the 5-day moving average of the daily deaths, and the black line represents the daily deaths calculated from the calibrated model. (b) Plots of the cumulative number of deaths versus different dates. The orange line in this plot shows Kerman's cumulative number of reported deaths, and the black line shows the cumulative number of deaths calculated from the calibrated model.

We calibrated our model to generate the epidemic trend in Kerman based on the observed deaths from February 4, 2020, to August 30, 2021 (Figure 2). Parameters were used from literature review and expert opinion, as well as Kerman and national empirical data. Contact rate, self-isolation rate, and average vaccination shots per day were changed based on the government interventions to observe the best fit between deaths in the model and real data. We used a Monte Carlo Markov Chain (MCMC) uncertainty analysis to obtain 95% uncertainty intervals (UI) around model estimates. We computed probability distributions for the model parameters, and 10,000 simulations were undertaken using a random sample. Therefore, for each day, we had 10,000 infected cases, deceased cases, and hospitalized cases. Moreover, the 2.5^th^ and 97.5^th^ percentiles and the mean of each 10,000 number were reported. MCMC uncertainty analysis was used during the process of calibration. More information about calibration is presented in the Supplementary Material (Appendix 2). The model parameters, definitions, values, and distribution are shown in Table 1 in the Supplementary Material (Appnedix 1).

### 2.3. Scenarios

We assumed that the contact rate was 13 before diagnosing the first case in Iran [37–39]. We calibrated the contact rate parameters based on the interventions implemented in Kerman. The average household size was considered as 4.3 in Kerman Province. Therefore, the minimum contact rate was estimated to be 5. Moreover, we assumed that 10% of patients self-isolated without any intervention. When Kerman's first case was diagnosed, self-isolation increased to 15% due to increased public awareness. After March 1^st^, the self-isolation rate increased due to patient self-reports through http://www.salamat.gov.ir/ and case finding through the 4030 system [6]. COVID-19 vaccination started in early February 2021. In the beginning, the average number of vaccination shots per day was approximately 120; however, it has increased to an average daily number of 13,000 people. We calibrated the contact rate, self-isolation rate, and average vaccination shots per day from February 4, 2020, to August 30, 2021, shown in Table 1.

In our model, six scenarios were presented, three of which predicted the total number of COVID-19 cases, deaths, and hospitalizations in Kerman from August 31, 2021, to December 31, 2021, (scenarios A, B, and C). For scenario A, the contact rate, self-isolation rate, and average vaccination shots per day were assumed to be 10, 30%, and 5,000, respectively (the worst-case scenario); for scenario B, the contact rate, self-isolation rate, and average vaccination shots per day were assumed to be 8, 40%, and 10,000, respectively (the moderate-case scenario); and for scenario C, the contact rate, self-isolation rate, and average vaccination shots per day were assumed to be 6, 50%, and 15,000, respectively (the best-case scenario). In these scenarios, contact rates, self-isolation rates, and average vaccination shots per day were modified to December 31, 2021.

Moreover, three scenarios (D, E, and F) examined the impacts of new COVID-19 variants and vaccination. In scenario D, we assumed that vaccination programs were not implemented with alpha and delta variants; in scenario E, we assumed that 70% of the population were vaccinated before the delta variant entered in Kerman (only with the presence of the alpha variant); in scenario F, we assumed that alpha, lambda, and delta variants were spread out simultaneously in June, and the vaccination rate was considered like the calibrated model. Our model was calibrated based on Kerman's death toll from February 04, 2020, to August 30, 2021. The interventions were changed during this period to evaluate new SARS-CoV-2 variants' impact and vaccination and compared with the calibrated model. The total number of COVID-19 cases, deaths, and hospitalizations were estimated under each scenario.

### 2.4. Time-Varying Reproductive Number (*R*_t_)

The basic reproduction number (*R*_0_) is the average number of secondary infections produced by an infected individual in a susceptible population during the infectious period. *R*_0_ is used when there is no immunity, vaccination, or intervention. The time-varying reproductive number (*R*_t_) is defined as the average number of secondary cases generated by primary cases when specified interventions (e.g., quarantine or isolation) are implemented. We calculated *R*_t_ with the time-dependent method based on the death toll in Kerman (see Appendix 3 in the Supplementary Material for more details). We used Vensim version 6.4 E software and R0 package in RStudio Desktop version 1.3.1093 to analyze the data.

## 3. Results

**Figure 3:**
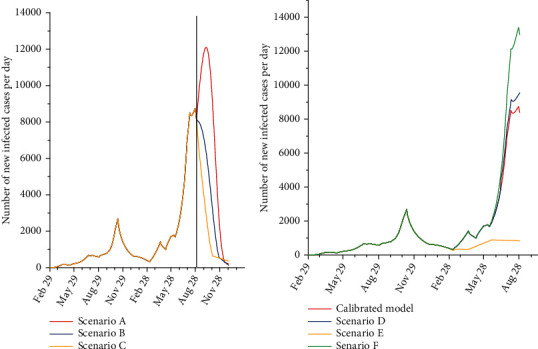
The estimated number of new infected cases per day in Kerman under different scenarios. ^∗^The black line shows the calibration for August 30, and each line is the mean of multiple runs.

**Figure 4:**
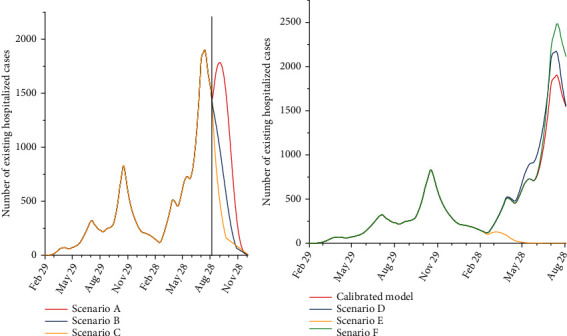
The estimated number of existing hospitalized cases in Kerman under different scenarios. ^∗^The black line shows the calibration for August 30, and each line is the mean of multiple runs.

**Figure 5:**
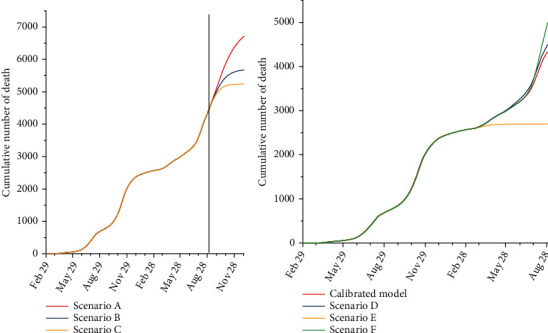
The estimated cumulative number of deaths in Kerman under different scenarios. ^∗^The black line shows the calibration for August 30, and each line is the mean of multiple runs..

Under scenario A (the worst-case scenario), it is anticipated that the total number of infected cases by December 31, 2021, would be 1,625,000 (95% UI: 1,112,000–1,898,000). Besides, the total number of hospitalized cases by December 31, 2021, is anticipated to be 83,000 (95% UI: 14,000–120,000). The total number of deaths is estimated to be 6,700 (95% UI: 5,200–8,700). Under scenario B (the moderate scenario), the total number of infected cases by December 31, 2021, is estimated to be 1,247,000 (95% UI: 815,000–1,583,000). The total number of hospitalized cases and death toll by December 31, 2021, is estimated to be 63,000 (95% UI: 11,000–95,000) and 5,600 (95% UI: 2,800–8,000), respectively. Under scenario F (the best-case scenario), the total number of infected cases by December 31, 2021, is expected to be 1,066,000 (95% UI: 686,000–1,347,000). The total number of hospitalized and death cases by December 31, 2021, is expected to be 55,000 (95% UI: 9,000–82,000) and 5,200 (95% UI: 2,000–7,500), respectively (Table 2). The estimated numbers of newly infected cases per day, existing hospitalized cases, and the number of deaths under different scenarios from February 29, 2020, to December 31, 2021, are shown in Figures 3–5.

It is predicted that the total number of infected cases under scenario D would be 957,000 (95% UI: 208,000–1,463,000). Under this scenario, the death toll and total number of hospitalized cases up to August 30, 2021, would be 4,500 (95% UI: 1,500–7,000) and 46,000 (95% UI: 10,000–70,000), respectively. Under scenario E, it is estimated that the total number of infected cases and the death toll up to August 30, 2021, would be 608,000 (95% UI: 122,000–743,000) and 2,700 (95% UI: 700–4,000). The total number of hospitalized cases in this scenario would be 31,000 (95% UI: 5,000–45,000). In scenario F, the total number of infected cases was expected to be 1,095,000 (95% UI: 255,000–1,800,000). It is estimated that the total number of death and the total number of hospitalized cases under scenario F would be 5,000 (95% UI: 1,500–7,500) and 54,000 (95% UI: 13,000–115,000), respectively (Table 3).

### 3.1. Time-Varying Reproductive Number (*R*_t_)

**Figure 6:**
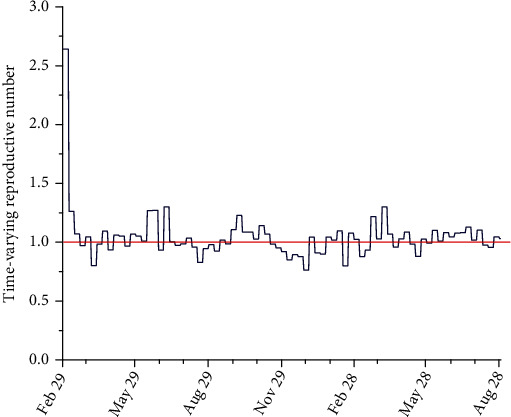
Time-varying reproductive number in Kerman, Iran, from 29 February 2020 to 30 August 2021.

We calculated *R*_t_ with a time-dependent method based on the number of deaths in Kerman. *R*_t_ was 2.6 when Kerman's first confirmed COVID-19-related deaths were reported in early March. It was reduced to <1 when strict limitations were implemented until mid-April. *R*_t_ was lower than one from mid-August to mid-September 2020 and from late November 2020 to late January 2021. Unfortunately, the trend of *R*_t_ has increased to ≥1 almost every day during the epidemic (Figure 6).

## 4. Discussion

We used an SEIR mathematical model to estimate the trend of the COVID-19 outbreak in Kerman, Iran. We also assessed timely vaccination programs, NPIs implemented by the government, and rate of self-isolation observed by patients and investigated the impact of three variants that were spread out simultaneously. The innovation of our study was the evaluation of three COVID-19 variants (alpha, delta, and lambda) simultaneously with and without vaccination programs. The results of this study suggested that if the vaccination programs were not implemented when alpha and delta variants spread out, the number of infected cases, hospitalizations, and deaths would have increased by 31%, 30%, and 28%, respectively. However, if 70% of the population were vaccinated before the delta variant entered Kerman (when only the alpha variant was dominant), the number of infected cases, hospitalizations, and deaths would have decreased by 87%, 95%, and 96%, respectively.

Unfortunately, due to the economic sanctions and the economic loss caused by the spread of COVID-19 in Iran, most economic interventions were gradually cancelled, and the number of COVID-19 cases has sharply risen in Iran [40, 41]. Suppression measures, highly effective contact tracing, and vaccination can reduce the disease burden. However, suppression measures have a devastating economic impact, particularly when preserved for a long time; therefore, it is urgent to enhance the rate of vaccination and contact tracing. Indeed, several studies have shown that the most effective way to diminish the transmission of COVID-19 is high vaccination coverage [42, 43]. Our results demonstrate that vaccination can reduce COVID-19-related mortality rate by around 90%, which aligns with vaccine effectiveness studies [44, 45]. Moreover, contact tracing and case isolation are other low-cost ways to control the COVID-19 epidemic. Hellewell et al. showed that COVID-19 could be controlled within three months via effective contact tracing and case isolation [26].

Studies have indicated that the new SARS-CoV-2 variants have significantly increased the risk of death and hospitalizations [28, 46–48]. Our results show that the three COVID-19 variants simultaneously increased the mortality and hospitalization rate by approximately 20% and 40%, respectively. To prevent the effect of new variants, vaccination of at least 70% of people could reduce deaths and hospitalization [49]. In addition to vaccination, other interventions, such as reducing social contacts, stricter limitations for gathering, public education to promote social distancing, incensing case finding and contact tracing, effective isolation, and quarantine, can reduce the transmission rate.

Moreover, we also predicted the number of deaths, infections, and hospitalizations in Kerman. In scenario A (worst-case scenario), the results indicate that more than 1,600,000 COVID-19 infections will occur in Kerman until December 31, 2021, of whom 5.1% will be hospitalized, and 0.41% will die. However, in the best-case scenario (i.e., scenario C), the number of infected, hospitalizations, and deaths could be reduced by 35%, 34%, and 23%, respectively. Based on scenario A (the worst-case scenario), the total number of existing hospitalized cases on September 18, 2021, was about 1,200 cases which is indeed very concerning given the availability of only 300 ICU beds and 150 ventilators to COVID-19 patients in Kerman Province.

In the current study, *R*_t_ decreased to <1 from mid-March to mid-April due to interventions enforced by public health agencies starting from February 19. However, after mid-April, *R*_t_ increased gradually, and it has been >1, which is consistent with other studies conducted in Iran [50, 51]. Also, our results show that without administration of vaccination and when the alpha and delta variants were dominant, *R*_t_ would be >1, and the epidemic would be out of control. The results are consistent with other studies which assess the impact of new COVID-19 variants and vaccines [27, 28]. These results highlight the importance of reducing *R*_t_ < 1 with improving vaccination coverage before other new COVID-19 variants appear.

We acknowledge the limitations of the study. First, some key parameters of the model, including the incubation period, hospitalization rate, and transmission probability, were derived from expert opinions or other countries due to Iran's unavailability of empirical data. To overcome this limitation, a range of uncertainty intervals were reported. Second, we considered some assumptions (e.g., the whole population was considered susceptible, assumed homogeneity of the susceptible and infectious population, and ignored migration rate between cities and the network topology in the model). Third, we could not present 95% uncertainty intervals in the figures because there was overlap between uncertainty intervals in different scenarios. If we wanted to report 95% uncertainty intervals, we had to report each scenario separately. Notwithstanding these limitations, the results of this study provided a foundation to evaluate the influence of interventions undertaken in Kerman under different scenarios.

## 5. Conclusion

Our data suggest that the most effective way to reduce the transmission of COVID-19 in Kerman is through high vaccination coverage. Also, the three COVID-19 variants simultaneously increased the risk of deaths and hospitalization rate by 20% and 40%, respectively. To prevent the effect of new variants, vaccination of at least 70% of people is required. Public education to promote social distancing, increasing daily tests, scaling up case finding, and contact tracing could reduce contact rate and increase self-isolation rate leading to a decline in the burden of COVID-19 in Kerman.

## Figures and Tables

**Table 1:** Calibrated contact rate, self-isolation rate, and the average vaccination shots per day before August 30, 2021, in Kerman, Iran.

Date	Calibrated model		
	Contact rate	Self-isolation rate (%)	Average vaccination shots per day
Feb 4, 2020, to Feb 19, 2020	13	10%	0
Feb 20, 2020, to Feb29, 2020	12	10%	0
Mar 1, 2020, to Mar 10, 2020	10	15%	0
Mar 11, 2020, to Mar 20, 2020	8	20%	0
Mar 21, 2020, to Apr 4, 2020	6	30%	0
Apr 4, 2020, to Apr 19, 2020	5	30%	0
Apr 19, 2020, to Apr 29, 2020	6	45%	0
Apr 30, 2020, to May 8, 2020	7	45%	0
May 9, 2020, to May 19, 2020	8	40%	0
May 19, 2020, to May 29, 2020	9	30%	0
May 30, 2020, to Jun 8, 2020	11	20%	0
Jun 9, 2020, to Jun 19, 2020	11	25%	0
Jun 20, 2020, to Jul 9, 2020	11	20%	0
Jul 10, 2020, to Jul 20, 2020	9	40%	0
Jul 21, 2020, to Jul 31, 2020	8	40%	0
Aug 1, 2020, to Aug 10, 2020	11	40%	0
Aug 11, 2020, to Aug 21, 2020	8	45%	0
Aug 21, 2020, to Sep 25, 2020	8	50%	0
Sep 26, 2020, to Oct 17, 2020	9	40%	0
Sep 29, 2020, to Nov 8, 2020	10	40%	0
Nov 8, 2020, to Jan 6, 2021	5	30%	0
Jan 7, 2021, to Feb 17, 2021	6	30%	120
Feb 18, 2021, to Mar 7, 2021	6	30%	120
Mar 8, 2021, to Apr 19, 2021	11	40%	120
Apr 20, 2021, to Apr 14, 2021	10	40%	1,000
Apr 15, 2021, to Jun 7, 2021	10	40%	1,000
Jun 8, 2021, to Jul 24, 2021	10	40%	200
Jul 26, 2021, to Aug 4, 2021	9	40%	4,000
Aug 5, 2021, to Aug 30, 2021	7	40%	13,000

**Table 2:** The estimated number of infected, hospitalized, and deaths to predict COVID-19 cases under three different scenarios from February 29, 2020, to December 31, 2021, in Kerman, Iran.

	Scenario A	Scenario B	Scenario C
Infected cases			
Total number (95% UI^∗^) until 31 December 2021	1,625,000 (1,112,000–1,898,000)	1,247,000 (815,000–1,583,000)	1,066,000 (686,000–1,347,000)
Hospitalized cases			
Total number (95% UI^∗^) until 31 December 2021	83,000 (14,000–120,000)	63,000 (11,000–95,000)	55,000 (9,000–82,000)
Deaths			
Total number (95% UI^∗^) until 31 December 2021	6,700 (5,200–8,700)	5,600 (2,800–8,000)	5,200 (2,000–7,500)

^∗^Uncertainty interval.

**Table 3:** The estimated number of infected, hospitalized, and deaths to assess the impact of new COVID-19 variants and vaccination under different scenarios from February 29, 2020, to August 30, 2021, in Kerman, Iran.

	Calibrated model	Reported data	Scenario D	Scenario E	Scenario F
Infected cases					
Total number (95% UI^∗^) until 30 August 2021	881,000 (202,000–1,375,000)		957,000 (208,000–1,463,000)	608,000 (122,000–743,000)	1,095,000 (255,000–1,800,000)
Hospitalized cases					
Total number (95% UI^∗^) until 30 August 2021	39,000 (9,000–67,000)	35,873	46,000 (10,000–70,000)	31,000 (5,000–45,000)	54,000 (13,000–115,000)
Deaths					
Total number (95% UI^∗^) 30 August 2021	4,300 (1,400–6,500)	4,263	4,500 (1,500–7,000)	2,700 (700–4,000)	5,000 (1,500–7,500)

^∗^Uncertainty interval.

## Data Availability

The data used to support the findings of this study are included in the manuscript.

## Supplementary Materials

Supplementary Materials The model parameters, definitions, values, and distribution are shown in Table 1 in Appendix 1. Information about calibration is presented in Appendix 2. The time-dependent reproductive number method based on epidemiologic data is provided in Appendix 3.
