# Supplementary material for: Estimating the Number of COVID-19 Cases and Impact of New COVID-19 Variants and Vaccination on the Population in Kerman, Iran: A Mathematical Modeling Study
Source: Comput Math Methods Med. 2022 Apr 26;2022:6624471. doi: 10.1155/2022/6624471 (PMC9039779; doi:10.1155/2022/6624471)
Supplement: Supplementary Materials — The model parameters, definitions, values, and distribution are shown in Table 1 in Appendix 1. Information about calibration is presented in Appendix 2. The time-dependent reproductive number method based on epidemiologic data is provided in Appendix 3. [file 6624471.f1.docx]

**Appendix 1**

The differential equations for each of the compartments are presented below:

$$\frac{\mathbf{dS}}{\mathbf{dt}}\boldsymbol{=-\beta}\left( \mathbf{t} \right)\mathbf{C}\left( \mathbf{t} \right)\frac{\mathbf{II}\left( \mathbf{t} \right)}{\mathbf{N}}\left( \mathbf{S}\boldsymbol{-}\boldsymbol{V}_{\boldsymbol{1}} \right)\boldsymbol{-}\left\{ \begin{aligned} \boldsymbol{\beta}\left( \mathbf{t} \right)\mathbf{C}\left( \mathbf{t} \right)\left\{ \left( \boldsymbol{V}_{\boldsymbol{1}}\boldsymbol{m}_{\boldsymbol{1}} \right)\boldsymbol{+}\left( \boldsymbol{V}_{\boldsymbol{2}}\boldsymbol{m}_{\boldsymbol{2}} \right) \right\}\frac{\boldsymbol{E}}{\boldsymbol{S}} \\ \boldsymbol{\beta}\left( \mathbf{t} \right)\mathbf{C}\left( \mathbf{t} \right)\left\{ \left( \boldsymbol{V}_{\boldsymbol{1}}\boldsymbol{m}_{\boldsymbol{1}} \right)\boldsymbol{+}\left( \boldsymbol{V}_{\boldsymbol{2}}\boldsymbol{m}_{\boldsymbol{2}} \right)\boldsymbol{+}\left( \boldsymbol{R}\boldsymbol{p}_{\boldsymbol{1}} \right)\boldsymbol{+}\left( \boldsymbol{R}\boldsymbol{p}_{\boldsymbol{2}} \right) \right\}\frac{\boldsymbol{E}}{\boldsymbol{S}} \end{aligned} \right. {\begin{aligned} \boldsymbol{Time<470} \\ \end{aligned} \atop\boldsymbol{Time>470}}$$

$$\frac{\mathbf{dE}}{\mathbf{dt}}\boldsymbol{=\beta}\left( \mathbf{t} \right)\mathbf{C}\left( \mathbf{t} \right)\frac{\mathbf{II}\left( \mathbf{t} \right)}{\mathbf{N}}\left( \mathbf{S}\boldsymbol{-}\boldsymbol{V}_{\boldsymbol{1}} \right)\mathbf{-}\frac{\mathbf{1}}{\boldsymbol{\delta}_{\boldsymbol{1}}}\mathbf{E}$$

$$\frac{\mathbf{d}\mathbf{E}_{\boldsymbol{1}}}{\mathbf{dt}}\mathbf{=}\left\{ \begin{aligned} \boldsymbol{\beta}\left( \mathbf{t} \right)\mathbf{C}\left( \mathbf{t} \right)\left\{ \left( \boldsymbol{V}_{\boldsymbol{1}}\boldsymbol{m}_{\boldsymbol{1}} \right)\boldsymbol{+}\left( \boldsymbol{V}_{\boldsymbol{2}}\boldsymbol{m}_{\boldsymbol{2}} \right) \right\}\frac{\boldsymbol{E}}{\boldsymbol{S}} \\ \boldsymbol{\beta}\left( \mathbf{t} \right)\mathbf{C}\left( \mathbf{t} \right)\left\{ \left( \boldsymbol{V}_{\boldsymbol{1}}\boldsymbol{m}_{\boldsymbol{1}} \right)\boldsymbol{+}\left( \boldsymbol{V}_{\boldsymbol{2}}\boldsymbol{m}_{\boldsymbol{2}} \right)\boldsymbol{+}\left( \boldsymbol{R}\boldsymbol{p}_{\boldsymbol{1}} \right)\boldsymbol{+}\left( \boldsymbol{R}\boldsymbol{p}_{\boldsymbol{2}} \right) \right\}\frac{\boldsymbol{E}}{\boldsymbol{S}} \end{aligned} \right. {\begin{aligned} \boldsymbol{Time<470} \\ \end{aligned} \atop\boldsymbol{Time>470}} \mathbf{-}\frac{\mathbf{1}}{\boldsymbol{\delta}_{\boldsymbol{1}}}\mathbf{E}_{\boldsymbol{1}}$$

$$\frac{\mathbf{dI}}{\mathbf{dt}}\mathbf{=}\frac{\mathbf{1}}{\boldsymbol{\delta}_{\boldsymbol{1}}}\mathbf{E-}\left( \frac{\boldsymbol{\theta}}{\boldsymbol{\delta}_{\boldsymbol{6}}}\mathbf{+}\frac{\boldsymbol{\alpha}}{\boldsymbol{\delta}_{\boldsymbol{8}}}\mathbf{+}\frac{\boldsymbol{\varepsilon}}{\boldsymbol{\delta}_{\boldsymbol{2}}}\mathbf{+}\frac{\boldsymbol{\omega}}{\boldsymbol{\delta}_{\boldsymbol{9}}} \right)\mathbf{I}$$

$$\frac{\mathbf{d}\mathbf{I}_{\boldsymbol{1}}}{\mathbf{dt}}\mathbf{=}\frac{\mathbf{1}}{\boldsymbol{\delta}_{\boldsymbol{1}}}\mathbf{E}_{\boldsymbol{1}}\mathbf{-}\left( \frac{\boldsymbol{\theta}_{\boldsymbol{1}}}{\boldsymbol{\delta}_{\boldsymbol{6}}}\mathbf{+}\frac{\boldsymbol{\alpha}_{\boldsymbol{1}}}{\boldsymbol{\delta}_{\boldsymbol{8}}}\mathbf{+}\frac{\boldsymbol{\varepsilon}_{\boldsymbol{1}}}{\boldsymbol{\delta}_{\boldsymbol{2}}}\mathbf{+}\frac{\boldsymbol{\omega}_{\boldsymbol{1}}}{\boldsymbol{\delta}_{\boldsymbol{9}}} \right)\boldsymbol{I}_{\boldsymbol{1}}$$

$$\frac{\mathbf{dR}}{\mathbf{dt}}\mathbf{=}\frac{\boldsymbol{\mu}}{\boldsymbol{\delta}_{\boldsymbol{5}}}\mathbf{T+}\frac{\boldsymbol{\alpha}}{\boldsymbol{\delta}_{\boldsymbol{8}}}\mathbf{I+}\frac{\mathbf{1}}{\boldsymbol{\delta}_{\boldsymbol{7}}}\mathbf{I}_{\mathbf{s}}$$

$$\frac{\mathbf{d}\mathbf{R}_{\boldsymbol{1}}}{\mathbf{dt}}\mathbf{=}\frac{\boldsymbol{\alpha}_{\boldsymbol{1}}}{\boldsymbol{\delta}_{\boldsymbol{8}}}\mathbf{I+}\frac{\mathbf{1}}{\boldsymbol{\delta}_{\boldsymbol{7}}}\mathbf{I}_{\mathbf{s}}$$

$$\frac{\mathbf{d}\mathbf{I}_{\mathbf{s}}}{\mathbf{dt}}\mathbf{=}\frac{\boldsymbol{\theta}}{\boldsymbol{\delta}_{\boldsymbol{6}}}\mathbf{I-}\frac{\boldsymbol{1}}{\boldsymbol{\delta}_{\boldsymbol{7}}}\mathbf{I}_{\mathbf{s}}$$

$$\frac{\mathbf{d}\mathbf{I}_{\mathbf{s1}}}{\mathbf{dt}}\mathbf{=}\frac{\boldsymbol{\theta}_{\boldsymbol{1}}}{\boldsymbol{\delta}_{\boldsymbol{6}}}\mathbf{I}_{\boldsymbol{1}}\mathbf{-}\frac{\boldsymbol{1}}{\boldsymbol{\delta}_{\boldsymbol{7}}}\mathbf{I}_{\mathbf{s1}}$$

$$\frac{\mathbf{dH}}{\mathbf{dt}}\mathbf{=}\frac{\boldsymbol{\varepsilon}}{\boldsymbol{\delta}_{\boldsymbol{2}}}\mathbf{I-(}\frac{\boldsymbol{\varphi}}{\boldsymbol{\delta}_{\boldsymbol{3}}}\mathbf{+}\frac{\boldsymbol{\rho}}{\boldsymbol{\delta}_{\boldsymbol{4}}}\mathbf{)H}$$

$$\frac{\mathbf{d}\mathbf{H}_{\boldsymbol{1}}}{\mathbf{dt}}\mathbf{=}\frac{\boldsymbol{\varepsilon}_{\boldsymbol{1}}}{\boldsymbol{\delta}_{\boldsymbol{2}}}\mathbf{I-(}\frac{\boldsymbol{\varphi}_{\boldsymbol{1}}}{\boldsymbol{\delta}_{\boldsymbol{3}}}\mathbf{+}\frac{\boldsymbol{\rho}_{\boldsymbol{1}}}{\boldsymbol{\delta}_{\boldsymbol{4}}}\mathbf{)}\mathbf{H}_{\boldsymbol{1}}$$

$$\frac{\mathbf{dT}}{\mathbf{dt}}\mathbf{=}\frac{\boldsymbol{\rho}}{\boldsymbol{\delta}_{\boldsymbol{4}}}\boldsymbol{H}\mathbf{-(}\frac{\boldsymbol{\mu}}{\boldsymbol{\delta}_{\boldsymbol{5}}}\mathbf{+}\frac{\boldsymbol{\tau}}{\boldsymbol{\delta}_{\boldsymbol{10}}}\mathbf{)T}$$

$$\frac{\mathbf{d}\boldsymbol{V}_{\boldsymbol{1}}}{\mathbf{dt}}\mathbf{=}\boldsymbol{\vartheta}_{\boldsymbol{1}}\left( \boldsymbol{S+R} \right)\mathbf{-}\boldsymbol{(\vartheta}_{\boldsymbol{2}}\boldsymbol{+}\boldsymbol{m}_{\boldsymbol{1}}\boldsymbol{)}\mathbf{V}_{\boldsymbol{1}}$$

$$\frac{\mathbf{d}\boldsymbol{V}_{\boldsymbol{2}}}{\mathbf{dt}}\mathbf{=}\boldsymbol{\vartheta}_{\boldsymbol{2}}\mathbf{V}_{\boldsymbol{1}}\boldsymbol{-}\left( \boldsymbol{V}_{\boldsymbol{2}}\boldsymbol{m}_{\boldsymbol{2}} \right)$$

$$\frac{\mathbf{dD}}{\mathbf{dt}}\mathbf{=}\frac{\boldsymbol{\varphi}}{\boldsymbol{\delta}_{\boldsymbol{3}}}\mathbf{H+}\frac{\boldsymbol{\omega}}{\boldsymbol{\delta}_{\boldsymbol{9}}}\mathbf{I+}\frac{\boldsymbol{\tau}}{\boldsymbol{\delta}_{\boldsymbol{10}}}\mathbf{T+}\frac{\boldsymbol{\varphi}_{\boldsymbol{1}}}{\boldsymbol{\delta}_{\boldsymbol{3}}}\mathbf{H}_{\boldsymbol{1}}$$

**Table 1:** Model parameters, definitions, values, distributions, and source of parameters used in the modified SEIR model

| **Parameters** | **Definition** | **Values** | **References** |
| --- | --- | --- | --- |
| C(t) | Contact rate is the mean of contacts between individuals, and it is time-varying | Values are reported in Table 2. | -- |
| $\beta$(t) | Transmission probability ($\beta$) is the probability of the infection being transmitted when an infected individual contacts a susceptible person. It is time-varying with a sine function. | Ranged from 0.045 in the winter to 0.02 in the summer  Seasonality distribution: Sin ((2 × 3.14 × (Time + 110)) / 365) + 1) × ((0.045 – 0.02) / 2)) + 0.02 | [1, 2] |
| II(t) | The potential of the infected individual transmitting the infection | I+(0.1×T)+(0.02×H) | -- |
| $\delta_{1}$ | The average duration between exposure and infection | Normal (5.33, 0.445)^a^ |  |
| m1 | Vaccine efficacy for people who received only one dose of vaccine | 50% | [3] |
| m2 | Vaccine efficacy for people who received two doses of vaccine | 70% | [3] |
| p1 | The proportion of recovered individuals who are re-infected with delta | Calibrated | -- |
| p2 | The proportion of recovered individuals who are re-infected with lambda | Calibrated | -- |
| $\delta_{6}$ | The [average time](https://www.powerthesaurus.org/average_time/synonyms) took an infected individual to observe self-isolation | Normal (3, 0.5)^a^ | Expert Opinion |
| $\delta_{7}$ | The average time took for an individual who is observing self-isolation to recover from the disease | Normal (7.91,0.5)^a^ | [4] |
| $\alpha$ | A proportion of infected individuals who recover without isolation or hospitalization | According to the possible scenarios | -- |
| $\alpha_{1}$ | A proportion of re-infected individuals who recover without isolation or hospitalization | According to the possible scenarios | -- |
| $\delta_{8}$ | The average time took an infected individual to recover without the need for isolation or hospitalization | Normal (10.91, 0.50)^a^ | [4] |
| $\varepsilon$ | The proportion of infected cases that are hospitalized | Normal (0.05, 0.01)^a^ | National Data and Expert Opinion |
| $\varepsilon_{1}$ | The proportion of re-infected cases that are hospitalized | Normal (0.05, 0.01)^a^ | National Data and Expert Opinion |
| $\delta_{2}$ | The average duration of hospital stay | Normal (2, 0.5)^a^ | Expert Opinion |
| $\rho$ | The proportion of hospitalized individuals who are discharged from the hospital and transferred to the temporary isolation unit | Normal (0.9, 0.01)^a^ | National Data and Expert Opinion |
| $\delta_{4}$ | The average time took hospitalized individuals to be discharged from the hospital | Normal (5, 0.5)^a^ | National Data and Expert Opinion |
| $\mu$ | the proportion of infected cases who recover after discharge | N(0.995, 0.001)^a^ | National Data and Expert Opinion |
| $\delta_{5}$ | The average duration of recovery after discharge | Normal (7, 0.5)^a^ | National Data and Expert Opinion |
| $\varphi$ | The proportion of hospitalized cases who die in the hospital | 1 – $\rho$ | -- |
| $\delta_{3}$ | The average duration of hospitalized persons who die in the hospital | Normal (5, 0.5)^a^ | Expert Opinion |
| $\omega$ | The proportion of infected cases who die from the infection without being hospitalized | 0.002 | Expert Opinion |
| $\omega_{1}$ | The proportion of re-infected cases who die from the infection without being hospitalized | 0.002 | Expert Opinion |
| $\delta_{9}$ | The average time in which the patient dies from the infection without being hospitalized | Normal (11, 0.50)^a^ |  |
| $\tau$ | The proportion of individuals who die from the infection after discharge | N(0.005, 0.001)^a^ | National Data Expert Opinion |
| $\delta_{10}$ | The average duration in which the infected person dies after discharge | Normal (7, 0.50)^a^ | Expert Opinion |
| $\vartheta_{1}$ | Average vaccination shot per day, and it is time-varying | Values are reported in Table 2. | -- |
| $\vartheta_{2}$ | The proportion of individuals who received two doses of vaccine | Calibrated | -- |

**Appendix 2**

In this study, we first determined some parameters based on the expert opinion, national data, and literature review. Then, we determined the distribution for some parameters using the MCMC method and generated 10,000 estimates from each of the parameters. We also calibrated three parameters based on government interventions implemented over time. These parameters were contact rate, self-isolation rate and average vaccination shots per day. As a result, we changed these three parameters based on the interventions to observe best fit between deaths in the model and observed data. For example, we knew that in the period Apr 4, 2020, to Apr 19, 2020, government implemented a quarantine policy. As a result, we knew that the number of contact rate should be between 4 and 6. And we also knew that in that period, the self-isolation rate in the Kerman was about 20% to 40%. Due to the lack of vaccines at that time, the average vaccination shots per day was zero. The model with the contact rate of 5, the self-isolation rate of 30% and the average vaccination shots per day of zero had the best fit compared to the real data in the period Apr 4, 2020, to Apr 19, 2020. Therefore, for some parameters, such as the proportion of hospitalized cases, we generated 10,000 iterations from the normal distribution with a mean of 0.05 and a variance of 0.01, some parameters such as contact rate changed over time, as shown in Table 1, and some parameters such as the proportion of infected cases who die from the infection without being hospitalized were also considered fixed. Finally, for each day, we had 10,000 infected cases, deceased cases, and hospitalized cases. Then, the 95% uncertainty intervals were taken as the 2.5^th^ and 97.5^th^ percentiles of the outputs and mean of each 10,000 number were calculated.

**Appendix 3**

**Time-dependent reproductive number method based on epidemiologic data**

In summary, the time-dependent method proposed by Wallinga & Teunis was calculated by averaging overall transmission networks compatible with the observed cases [5, 6].

Time-varying R_t_ can be estimated by time-dependent with formula R_t_=$\frac{1}{N_{t}}\sum_{t_{j}=t} R_{j}$ where $R_{j}=\sum_{i} P_{i}$ and $P_{ij}=\frac{N_{i}w(t_{i}-t_{j})}{\sum_{i\neq k} N_{i}w(t_{i}-t_{k})}$. Where, Pij shows the probability of infection transmission from case i (in time ti) to case j (in time tj). R_t_ is the mean of all Rj computed by all networks of observed cases. Also, we considered gamma distribution for serial intervals with the mean and the standard deviation of 4.55 and 3.3 days, respectively [7].

**References**

1. Wan H, Cui JA, Yang GJ, “Risk estimation and prediction of the transmission of coronavirus disease-2019 (COVID-19) in the mainland of China excluding Hubei province,” Infectious Diseases of Poverty, vol. 9, pp. 1–9, 2020. doi:10.1186/s40249-020-00683-6.

2. Haghdoost A.A, Gooya M.M, Baneshi M.R, “Modelling of H1N1 flu in Iran,” *Archives of Iranian Medicine, vol. 6, pp. 533-541, 2009.*

3. How do China’s COVID vaccines fare against the Delta variant? <https://fortune.com/2021/08/31/china-covid-vaccine-sinovac-sinopharm-delta-variant-effective/>

4. You C, Deng Y, Hu W, “Estimation of the time-varying reproduction number of COVID-19 outbreak in China,” *International Journal of Hygiene and Environmental Health*, vol. 228, pp. 113555, 2020. doi:10.1016/j.ijheh.2020.113555

5. Wallinga J, Teunis P, “Different epidemic curves for severe acute respiratory syndrome reveal similar impacts of control measures,” *American Journal of epidemiology*, vol. 160, pp. 509-516, 2004. doi:10.1093/aje/kwh255.

6. Cauchemez S, Boëlle PY, Donnelly CA, et al., “Real-time estimates in early detection of SARS,” *Emerging infectious diseases*, vol. 12, pp.110, 2006. doi: 10.3201/eid1201.050593.

7. Aghaali M, Kolifarhood G, Nikbakht R et al., “Estimation of the serial interval and basic reproduction number of COVID‐19 in Qom, Iran, and three other countries: A data‐driven analysis in the early phase of the outbreak,” *Transboundary and emerging diseases*, vol. 67, pp. 2860-2868, 2020. doi: 10.1111/tbed.13656
